# Assessment of brain cancer atlas maps with multimodal imaging features

**DOI:** 10.1186/s12967-023-04222-3

**Published:** 2023-06-12

**Authors:** Enrico Capobianco, Marco Dominietto

**Affiliations:** 1 The Jackson Laboratory, 10 Discovery Drive, Farmington, CT 06032 USA; 2 Paul Scherrer Institute (PSI), Forschungsstrasse 111, 5232 Villigen, Switzerland; 3 Gate To Brain SA, Via Livio 7, 6830 Chiasso, Switzerland

**Keywords:** GBM, MRI imaging, Brain cancer atlas, Radiomics, Multimodal integration

## Abstract

**Background:**

Glioblastoma Multiforme (GBM) is a fast-growing and highly aggressive brain tumor that invades the nearby brain tissue and presents secondary nodular lesions across the whole brain but generally does not spread to distant organs. Without treatment, GBM can result in death in about 6 months. The challenges are known to depend on multiple factors: brain localization, resistance to conventional therapy, disrupted tumor blood supply inhibiting effective drug delivery, complications from peritumoral edema, intracranial hypertension, seizures, and neurotoxicity.

**Main text:**

Imaging techniques are routinely used to obtain accurate detections of lesions that localize brain tumors. Especially magnetic resonance imaging (MRI) delivers multimodal images both before and after the administration of contrast, which results in displaying enhancement and describing physiological features as hemodynamic processes. This review considers one possible extension of the use of radiomics in GBM studies, one that recalibrates the analysis of targeted segmentations to the whole organ scale. After identifying critical areas of research, the focus is on illustrating the potential utility of an integrated approach with multimodal imaging, radiomic data processing and brain atlases as the main components. The templates associated with the outcome of straightforward analyses represent promising inference tools able to spatio-temporally inform on the GBM evolution while being generalizable also to other cancers.

**Conclusions:**

The focus on novel inference strategies applicable to complex cancer systems and based on building radiomic models from multimodal imaging data can be well supported by machine learning and other computational tools potentially able to translate suitably processed information into more accurate patient stratifications and evaluations of treatment efficacy.

**Graphical Abstract:**

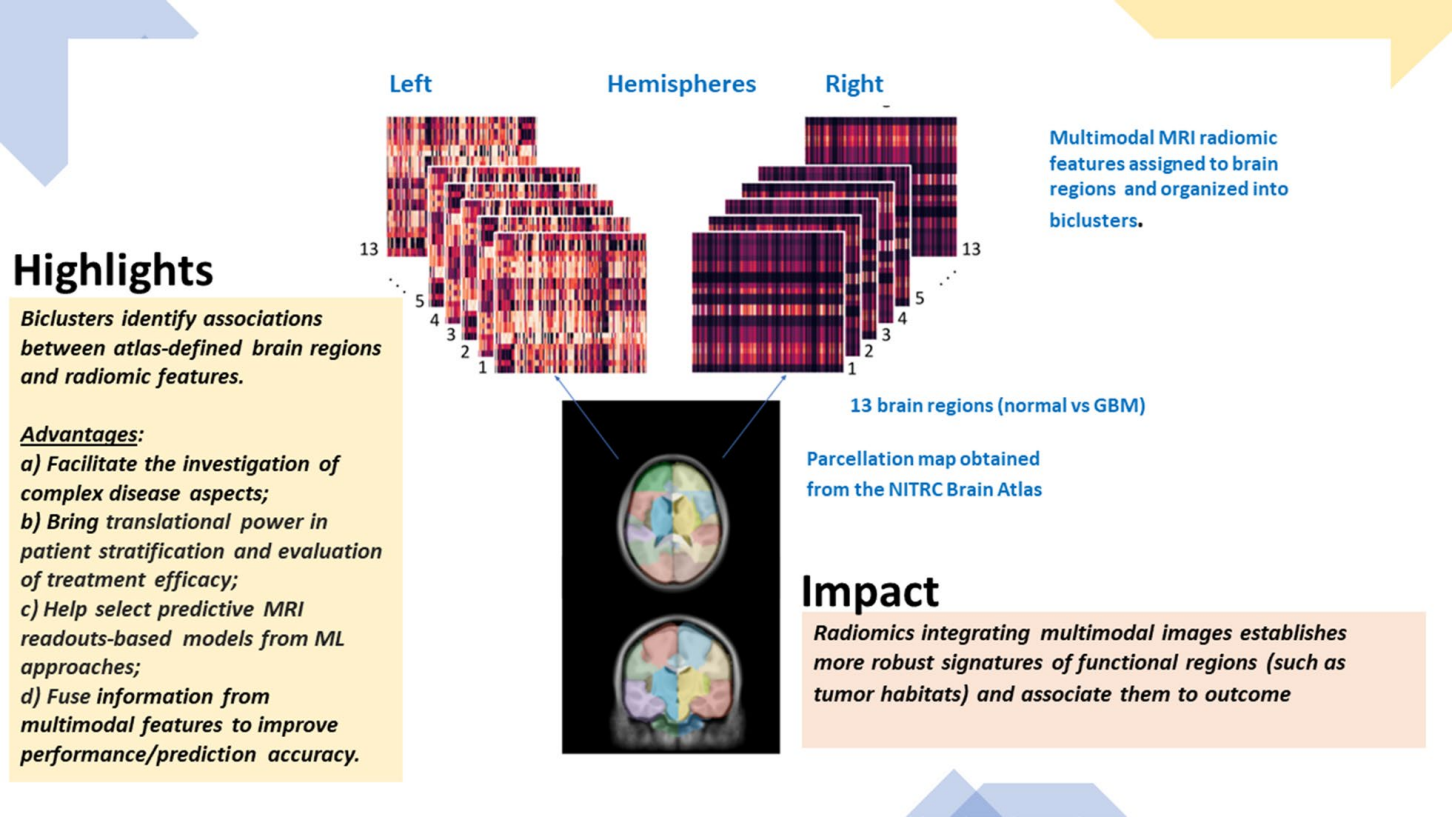

**Supplementary Information:**

The online version contains supplementary material available at 10.1186/s12967-023-04222-3.

## Introduction

### Glioblastoma multiforme (GBM)

GBM is the most common type of malignant primary brain tumor but is still not well understood. It presents a poor prognosis with a median survival of about 15 months for patients who receive standard therapy (Stupp protocol, based on surgical resection and radiotherapy combined with concomitant chemotherapy). Population-RWD (real-world data) based meta-analyses suggest that 2-, 3- and 5-year survival in GBM patients only partially improved since 2005 [1]. Recent advances in understanding the molecular biology of GBM leveraged studies on genetic alterations and genomic profiles [2–4], including subclone diversity, via single cell DNA sequencing, and transcriptome profile diversity, via single cell RNA-sequencing [5, 6].

In common with other cancers, the tumor microenvironment (TME) plays a complex role in GBM too. Generally responsible for growth and invasion, TME is highly variable at the intra-tumor spatial level, and both cellular and molecular interactions have not yet led to causal explanations [7, 8]. However, the increased imaging centrality driving radiomic studies, especially magnetic resonance imaging (MRI) in GBM, has drawn novel interest in computational modeling to improve diagnosis, prognostication and clinical decision support [9–11].

## Radiomics

Radiomics is a multidisciplinary field that engages scientists in processing various interconnected tasks such as tumor segmentation, image preprocessing, feature extraction, model development, testing and validation. Usually, segmentation is performed on annotated images by targeting whole tumors, subregions or peritumoral areas (all defined through regions of interest or ROI) to extract features describing distributions of signal intensities and spatial relationships. Selected features are those bringing differentially informative content regarding texture, shape, statistical descriptions, intensity-based measures etc. The major interest is in texture, as it reveals patterns characterized by brightness, color, slope, size. The analysis of features aims to quantify variations in intensity values and gray levels. Other relevant tumor information comes from shape characteristics whose analysis elucidates geometric aspects through shape descriptors.

There are a few factors in the radiomics workflow that influence these features and their significance. First, features may undergo manual, semi-automatic or automatic treatment. For instance, automatic learning typically involves Machine Learning (ML) and Deep Learning (DL) algorithms that tend to outperform more classical statistical techniques with complex and big datasets [12–14]. The search for feature saliency may require a reduction of dimensionality to embed only the most informative characteristics then used to train models and determine the best possible accuracy. Alternatively, analyses supported by hand-crafted features may also be highly valuable due to superior interpretability [15, 16], even when combined with ML-derived ones [17–19]. This choice might be instrumental to clinical translational, given multiple criteria to be applied [20, 21].

Due to the substantial image data space that has become available through data repositories for research, examples being The Cancer Imaging Archive, TCIA (https://www.cancerimagingarchive.net/) [22] or the newborn EuCanImage (https://molgenis.eibir-edc.org/), there is a massive call for analysis based on computational and predictive modeling and developments of novel integrative multi-omics inference tools. The applications range from tumor segmentation and anatomical lesion detection [23] to computer-aided diagnosis and prognosis [24, 25], just to mention a few. Some criticism persists relatively to a certain lack of interpretability [26], nonetheless inspiring the emerging explainable artificial intelligence (XAI) [27–30] research. In general, despite the necessity to measure any radiomic tool in terms of clinical value, our understanding of intra-tumor heterogeneity in GBM has received a strong impulse from radiomics with reference to a few specific domains. No doubt that a better characterization of intra-tumor heterogeneity will determine any future progress in terms of outcome prediction and personalized treatment.

### Focus areas

#### TME, habitats and saliency

As tumor regions are biologically different, the most specific use of radiomic features is one that leverages these quantitative measures to support genetic or epigenetic evidence as well as phenotypes (aggressiveness degree, resistance mechanisms, etc.) elucidating differentiated patterns of metabolism, hypoxia, proliferation, neovascularization. Radiomics that informs on tumor surrounding tissues, including the TME, provides additional prognostication power (for instance, in high-grade glioma [31, 32] this affects the possibility to predict tumor’s aggressiveness) and ability to monitor patient’s response to therapy via follow-up imaging and detect new or modified areas of enhancement, e.g., assessment of risk of tumor progression (TP) versus treatment-related changes or pseudo-progression (PsP) [33, 34].

One aspect of heterogeneity in tumors consists of differentiated TMEs, something which has motivated the recent investigation of the so-called habitats [35–40]. Imaging habitats can quantify the grey-level heterogeneity appearing from scans, which helps detect variations in tumor blood supply. Generally obtained by supervised segmentation of the tumor into sub-regions that map its complex organizational structure, the complexity of habitats naturally translates into a very rich data milieu needing ad hoc computational treatment, even beyond the association of habitats with somehow defined clusters [41]. Clustering methods can identify tumor sub-regions contributing with varying prognostic performances. However, the identified clusters or sub-regions are usually the result of the application of globally defined thresholds that often have minimal biological value. Also, biology may not be able to guide selectively the cluster being formed or a reasonable weighting based on the retrieved radiomics features. Finally, clustering often employs simple structures determined with some degree of arbitrariness (say, the choice of the number of clusters) or may assume data hierarchies that are algorithmically valid but have limited contextual relevance (say, random forest, trees etc.).

All the above aspects influence the definition of saliency, for instance relatively to the hypotheses about the importance to assign to tumor regions based on their phenotypic contributions. In principle, a clinically useful saliency map would be predictive for the risk of relapse given the improved model performance expected when selected (salient) features are chosen. However, each specific context must be carefully analyzed, and for instance GBM has a highly complex background with lots of redundant information due to its the multiscale nature. This somehow contrasts with the idea of targeting specific ROI embedding the saliency of contextual characteristics, and is something not linearly solvable (i.e., by fixing thresholds to establish saliency that identify significant foci or connecting to them surrounding regions through some functions or distances).

### Multimodal data integration

To address saliency, it is important to consider the fact that imaging informs at the spatial level by identifying sub-regions that may vary across multi-modal sequences (e.g., T1, T1-post contrast, T2 and FLAIR sequences etc.). Interestingly, multi-modality extends beyond the imaging combinations to include the associations with phenotypes, i.e., gene expressions and correlation with molecular subtypes [42], or outcome via survival. These developments have inspired the field of radiogenomics revealing associations between imaging phenotypes (tumor location, neo-angiogenesis, tumor enhancement etc.) and molecular marks, ultimately leading to refined patient stratifications [43–45]. Also, the differentiation of tumor molecular profiles based on imaging traits implies that identified MRI phenotypes may be used to probe the underlying genotypes [46–48]. MRI-driven radiomics has probed substantial relationships also between genomic GBM profiles and imaging traits [49, 50].

Leveraging imaging multi-modality implies taking decisions on what modes to combine and why, based on the general expectation of building more effective radiomic models compared to those dependent on any single modality only. Decisions face a spectrum of possible combinations of different MRI sequences [51], with relevance assigned to their clinical impact [52]. At data and computational levels, the fusion of multiple modalities may also vary, for instance depending on the stage. At early stages, i.e., before feature classification, when combined with clinical information (risk factors etc.). Inherent technological aspects may also prevail, for instance with CT presenting insufficient soft-tissue contrast. More in general, one problem is the presence of imbalanced classes in the data sets, something affecting the classifier learning ability, likely inducing bias towards the majority class, incorrect predictions and less robust models.

Potential gains from modality fusion in terms of model performance can be tested both during the modeling stage, when fusions may depend on active or incremental learning, and during later stages (decision level) too. [53–56]. Data heterogeneity is another aspect, as the fusion of mixed data types should achieve superior predictive accuracy. In both such regards, model performance improvements and data diversity, the role of ML toward handling large amounts of radiomic features characterizing tumor phenotypes integrated with other data types has primarily focused on building accurate and replicable models for tumor classification and outcome prediction. As an example, multimodal MRI radiomics can effectively differentiate GBM from lower grade gliomas and characterize the IDH and 1p/19q status using a ML approach useful to clinical practice [57]. In general, treating multimodality presents several complexities. One is inherent to the definition of multimodality (restricted to imaging or inclusive of other data types) [58]. Another is more contextual and may refer for instance to type of treatment and disease evolution [59]. Then, while more robust estimations can be reasonably expected with multimodal methods, scalability can be an issue, although deep neural networks show high accuracy with hyper-scale data sets (about 5 ml images) [60].

Finally, another interesting domain linked to both habitats and data integration/multimodality is imaging synthesis [61–65], where an increased number of image features retrieved from multiple regions and different sources may require relatively large cohort sizes. This may offset the limited biological meaning found in habitats by leveraging large-scale validations involving tissue phenotypes and histology. Note that spatial co-registration of images and histology can be instrumental to the use of MRI habitats for delineation of hypoxia, necrosis etc. [66]. Especially with reference to necrosis, significant contributions have come from multiple studies [67–72], indicating the informativeness and classification impact of both handcrafted and deep features extracted from multimodal MRI images. Of note that radiomic texture features informative about both the lesion and the peri-lesion environment (defined as “lesion habitat”) can distinguish radiation necrosis and tumor recurrence [73].

### Pillars

#### Brain atlases as integrative tools

Brain function is related to brain organization, which in turn refers to spatial heterogeneity. Brain Atlases usually operationalize these concepts through the parcellation or partitioning of the brain in multiple closely interacting regions [74]. Many atlases exist that collect brain data (tumor and non) and describe high-resolution location-specific maps centered on imaging data, becoming tools that support quantitative analyses. For instance, fMRI or functional MRI-driven brain studies help define probabilistic maps based on functional and structural data [75]. In general, non-random localizations of lesions are identified through specific patterns and marks in capture points or ROI [76]. Then, brain’s information is processed from characteristics at both structural and functional levels [77]. A common goal is to infer causal influences from experimental measures and exploit the interconnectivity between multiple data to provide mechanistic insights on the nature of their relationships. While fMRI estimates functional connectivity (brain activity), the discovery of brain disease signatures emerging from different brain network patterns can also result from integrating other MRI types, e.g., diffusion (dMRI) and structural (sMRI) to study anatomical and pathological connectivity from shape, size, and integrity of brain structures.

Atlases can be useful reference tools and represent templates for brain segmentation tasks. Among the many available resources and tools, *The neuromaps software toolbox* [78] (https://github.com/netneurolab/neuromaps) offers structural and functional annotations of the human brain through a variety of reference maps and biological ontologies. Then *Neuroparc* [79] (https://github.com/neurodata/neuroparc), conceived to standardize existing atlas repositories (46 different adult human brain parcellations of various type, surface-based, volume-based etc.). Global integrative analyses may require other types of tools such as *BCGene* [80] (http://soft.bioinfo-minzhao.org/bcgene/), which explores genetic mechanisms from about 1400 literature-curated human genes in 40 brain cancer subtypes and about 3000 patients. Specific to GBM, a well-known resource is the Ivy Glioblastoma Atlas Project (Ivy GAP) [81, 82] (http://glioblastoma.alleninstitute.org/), a comprehensive resource on GBM anatomy and genetics characterizing the cellular and molecular structures.

### Connectome for functional studies

Connectome research [83, 84] is an area potentially receiving a strong impulse from brain atlases. A reference study [85] in brain tumor connectome analyzed neuroimaging data from 335 adult patients with high- and low-grade glioma and combined them into a replicable tumor frequency map correlated with multiple graph-theoretical metrics establishing high functional connectedness. The application of a regression model with connectome, cellular, and genetic variables has explained 58% of the variance in glioma frequency, showing the independently exerted influences over the anatomic localization of oncogenesis. Another study [86] has leveraged the mapping of independent sources of glioma localization determining their relationships with neurogenic niches, genetic markers, and large-scale connectivity networks. Then, by applying independent component analysis (ICA) to lesion data from 242 adult patients with high-/low-grade glioma, three lesion covariance networks were identified to represent clusters of frequent glioma localization. These networks were associated with clinical variables and genomic information, and structural/functional connectivity was derived from neuroimaging data to uncover brain networks prone to tumor development.

### Neuroplasticity

Research on GBM radiomics requires precise definition of structural and functional features to infer factors explaining pathogenetic mechanisms together with disease progression or evolution [87]. By combining such features, MRI technologies can explore the neuroplasticity of structural, topological, biochemical metabolism, and related mechanisms [88]. MRI is typically used to provide detailed anatomical and pathological information in addition to physiological detail. Neuroplasticity caused by highly heterogeneous brain tumors could benefit from multimodal MRI offering individualized prediction of functional prognosis of patients based on ML algorithms [89]. Through the (semi-) automatic identifications of image features, ML brings accuracy in the classification and facilitates the integration with molecular profiles, histological tumor grade, and prognostic factors by using images acquired both at diagnosis and treatment, including follow up to enable differentiation between response and post-treatment-related effects.

### Brain atlas and radiomics integration

To demonstrate the potential use of brain atlases in combination with radiomics, we chose the atlases and databases publicly available from UNC-Chapel Hill (as part of NITRC, ‘NeuroImaging Tools & Resources Collaboratory’, https://www.nitrc.org/projects/unc_brain_atlas), and chose ‘UNC_Adult_Brain_Atlas_1’, i.e., the atlas of normal adult human brain anatomy generated from 50 + healthy adult cases (20–59 years old). The atlas comes with T1-weighted images (with and without skull), tissue segmentation probability maps (white matter, gray matter, CSF, rest) and a 27-lobe parcellation map.

The radiomic data were obtained from the multimodal MRI of GBM samples and TCIA radiological data (clinical images) matched to TCGA subjects, considering only a couple of patients (for demonstrative scopes). We subjected the samples to standard open source tools, i.e., 3D Slicer (https://www.slicer.org/) and pyradiomics [90], https://www.radiomics.io/pyradiomics.html. The details of the data sets from The Cancer Genome Atlas Glioblastoma Multiforme Collection (TCGA-GBM) are available at https://wiki.cancerimagingarchive.net/display/Public/TCGA-GBM, and they aim to connect radiological phenotypes to tissue genotypes and patient outcomes. Note that the collected data focus on routine care rather than controlled studies or clinical trials, tissues from multiple sites and heterogeneous images due to different scanner modalities and acquisition protocols.

**Fig. 1:**
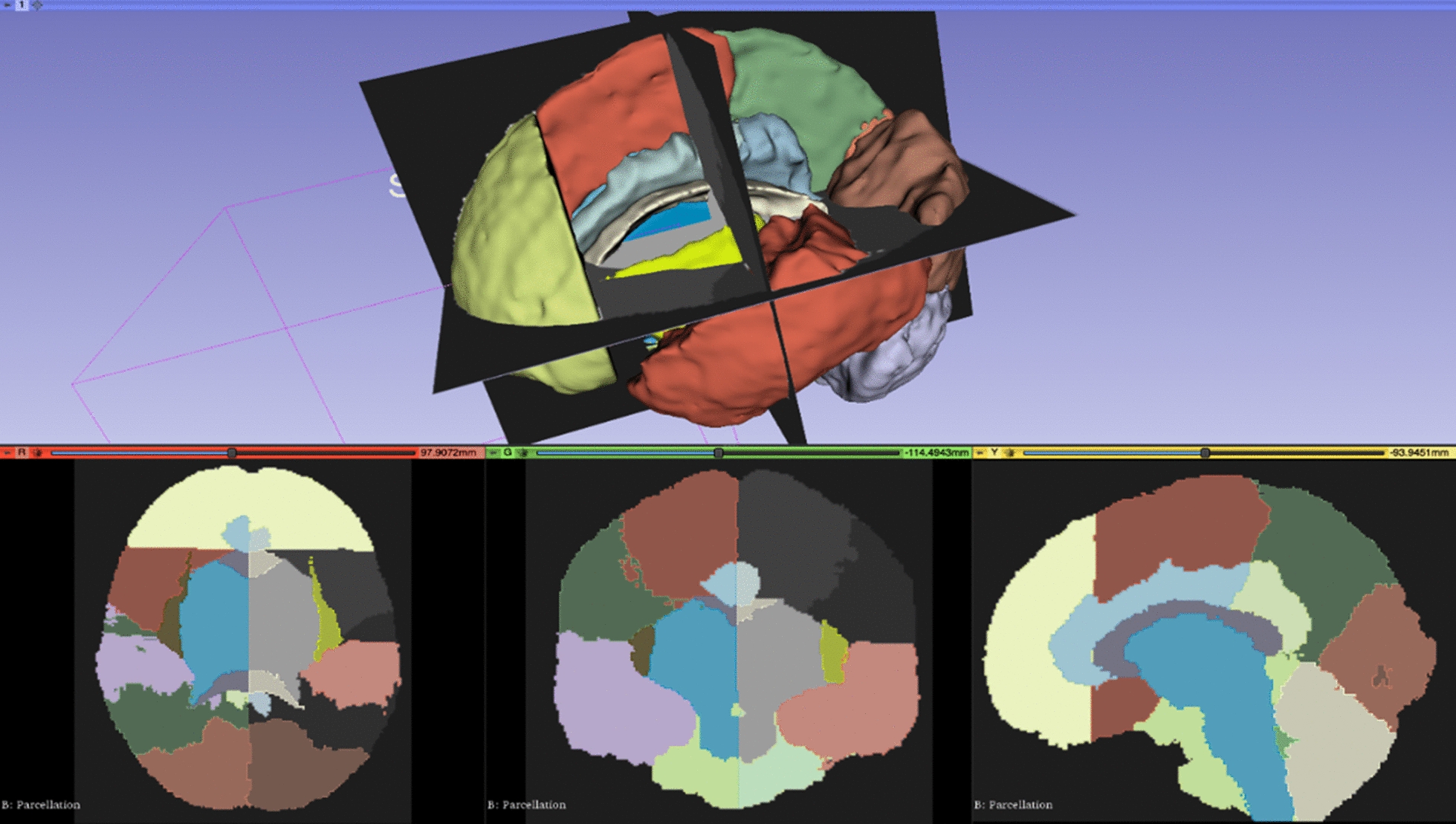
Parcellation map obtained from the NITRC Brain Atlas (https://www.nitrc.org/)

**Fig. 2:**
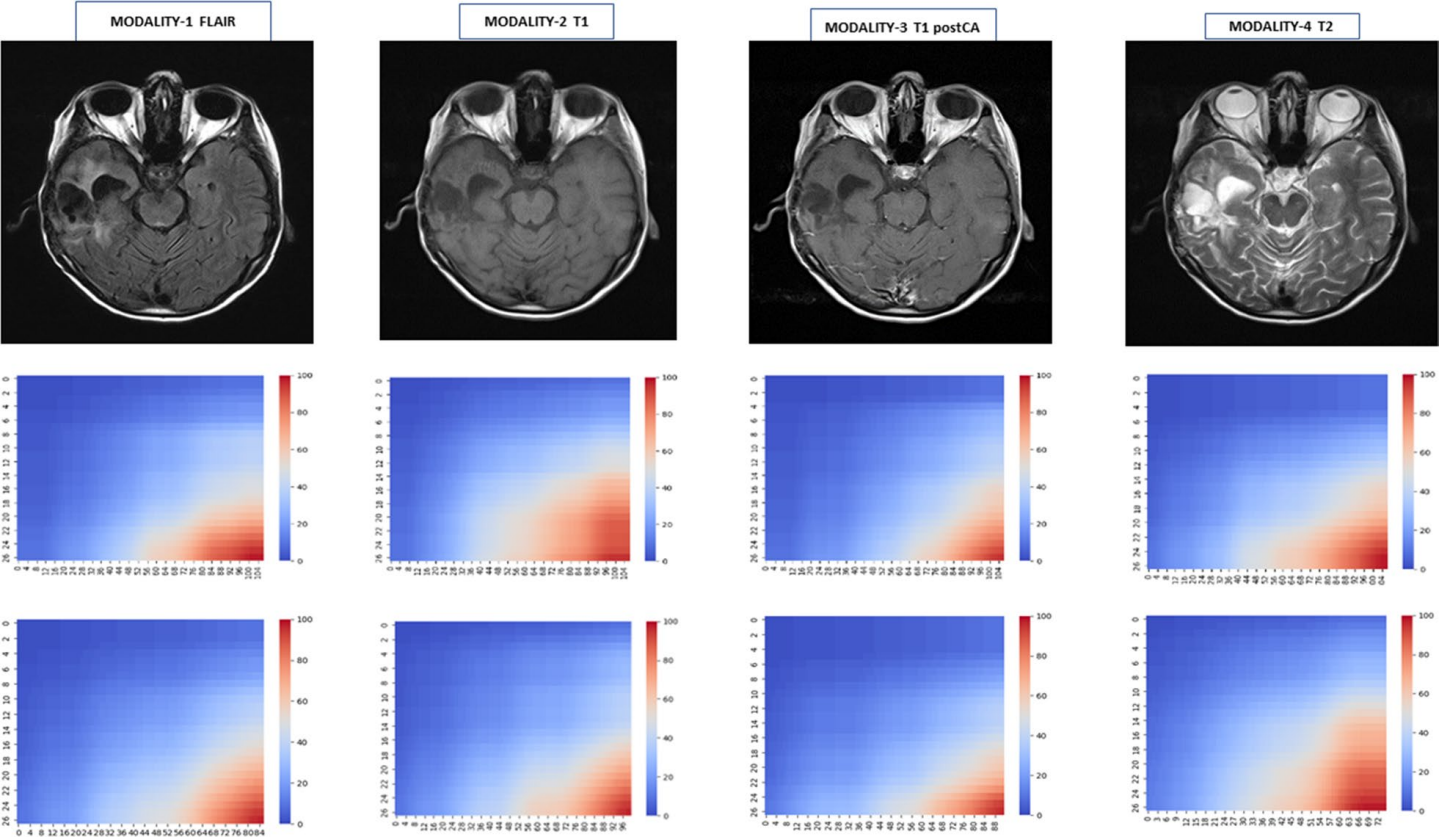
MRI modalities (top) and Biclustering maps. For one GBM patient, four MRI modalities support biclustering maps displayed with all features (centre) or selected ones (bottom). The applied spectral biclustering algorithm https://scikit-learn.org/stable/modules/biclustering.html [91] assumes a hidden checkerboard structure for the input matrix and thus partitions its rows and columns according to a blockwise-constant checkerboard matrix. The most significant Gauss filtered biclusters appear at the bottom-right corner

**Fig. 3:**
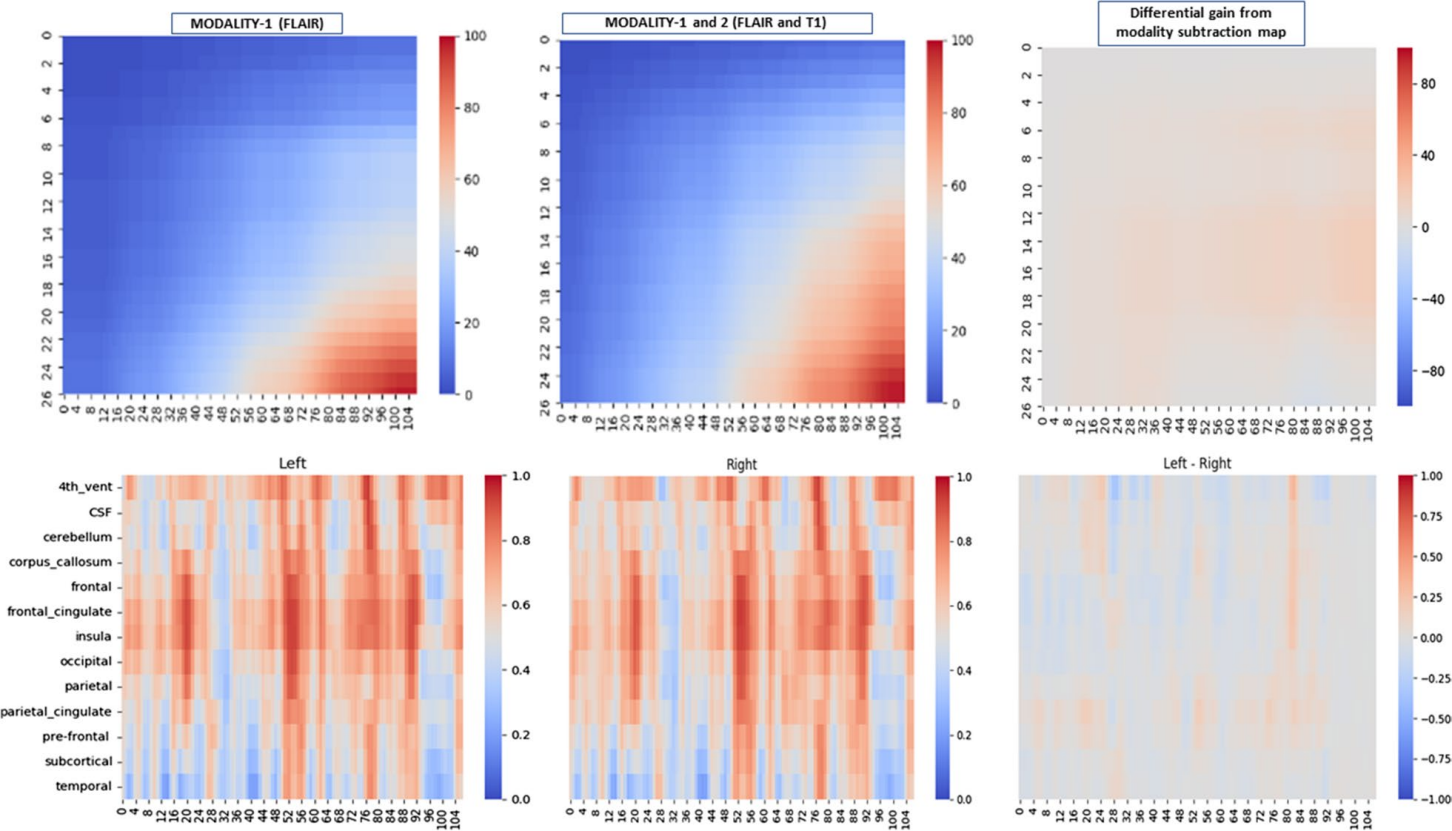
Top: Combined MRI modalities in Gauss filtered bicluster maps. Left: single modality. Centre: two combined modalities. Right: effects of modality subtraction (from two to one). Bottom: Visualization of differential effects induced by GBM in left and right hemispheres (N = 13 regions used to map) and by subtracting unsorted biclustering maps (rightmost plot). The MRI modality used is FLAIR

The visualizations of Fig. 1 display examples of parcellation of the normal brain regions. By co-registering the frame that the atlas parcellation provides and the GBM images across the MRI modalities, we obtained spatially matched and integrated brain maps covering both normal and tumor brain regions. Single and combined MRI characteristics were then mapped (Figs. 2 and 3, respectively) also considering the two brain hemispheres (with and without GBM) for measuring differential effects.

The visual associations between brain regions with or without GBM signatures retrieved from MRI radiomic features is well represented by biclusters. We looked for differential effects captured by the different MRI modalities (modality-1 = ‘FLAIR’; modality-2 = ‘T1w’; modality-3 = ‘T1w postCA’; modality-4 = ‘T2w’). In principle biclustering can identify associations between atlas-defined brain regions and radiomic features, offering a few advantages: (a) Facilitating the investigation of complex disease aspects (pseudo-progression and other treatment-related effects); (b) Bringing translational power for patient stratification and evaluation of treatment efficacy when combined with clinical information; (c) Help select predictive MRI readouts-based models from ML approaches and libraries and use the fusion of information from multimodal features to improve the model performance in terms of prediction accuracy.

Some limitations may apply. Radiomics performs well by training over large data sets with accurate labels (from expert annotations) to facilitate the process of reproducing results of predictive models, generalizing across multiple training data sets, validating over independent patient cohorts and ultimately translating into the clinics. Nevertheless, radiomic methods that integrate multimodal images can establish robust signatures of functional regions (such as tumor habitats) and associate them to outcomes.

By looking at Table 1 and considering that these annotations refer to the top-scored biclusters in Fig. 2 (bottom-right corner), the imaging results in patient A show three recurrent brain regions (l = left, r = right), i.e., *l_parietal, r_frontal* and *r_parietal_cingulate* across three modalities, i.e., FLAIR, T1w, T2w. Then 2 regions, *l_CSF* and *r_prefrontal* informed by T1w and T2w. In patient B, *r_parietal_cingulate* recurs across FLAIR and T1w, and *r_corpus_callosum, l_cerebellum*, *r_subcortical, l_*temporal recur across T1w postCA and T2w. At the opposite, *r_cerebellum* and *l_subcortical* appear only in patient B, while *r_frontal* only in patient A. Interestingly, very different radiomic feature combinations appear in correspondence with the modalities for the two patients (see the Additional file 2 ‘SM_Allregions’ with all the atlas brain regions).

The FLAIR modality indicates the relevance of texture aspects more in Patient A than B, although with a limited overlap of brain regions involved. T1w informs on new brain regions from both patients and includes similar features with respect to local homogeneity properties of the images. T1w postCA involves a mix of brain regions in part overlapping between the two patients but associated to different features referred to shape descriptors (from ROI in both 2D and 3D) and texture heterogeneity (via glcm or gray level co-occurrence matrix, i.e., second-order joint probability function of image regions). Finally, T2w informs in Patient A on three brain regions seen with T1w but associated to totally different features, relatively to texture. In Patient B some similarities emerge from brain region overlaps with Patient A and previously informed by T1w postCA modality through texture-related features, but here additionally related to shape features (see Additional file: 1 ‘SM_ALLfeatures’ with all the listed features, and for the source details https://pyradiomics.readthedocs.io/en/latest/features.html).

**Table 1:** Biclusters from 2 patients across all MRI modalities combine brain regions and radiomic features. Note that for reasons of space only the data of patient A was previously visualized in Fig. 2

	Brain regions	Radiomic features
Patient A		
Modality-1 FLAIR	2, 5, 22	8, 10, 14, 19, 35, 40, 49, 60, 71
Modality-2 T1w	2, 5, 22, 25, 26	1, 17, 28, 29, 34, 58, 74
Modality-3 T1w postCA	1, 5, 12, 13	97, 99, 100, 101, 103
Modality-4 T2w	22, 25, 26	11, 33, 67, 68, 70, 82, 83, 92
Patient B		
Modality-1 FLAIR	14, 15, 22	3, 16, 18, 20, 48, 59, 75, 76, 79, 81, 84, 102, 105, 107
Modality-2 T1w	2, 22	1, 8, 10, 28, 29, 34
Modality-3 T1w postCA	12, 13, 16, 17	15, 24, 30, 31, 32, 43, 86
Modality-4 T2w	12, 13, 16, 17, 25, 26	12, 23, 27, 82, 83, 91, 94, 95, 104

### Discussion and concluding remarks

We have reviewed GBM and some emerging research topics that hopefully can advance the field in the immediate future. Among the identified needs that require pre-clinical investigation, there are integrative and multimodal imaging data approaches. These are especially associated in brain to imaging tools such as MRI that offer multiparametric solutions and interpretations.

As examples of emerging concepts falling into these new developments there are imaging habitats that capture tumor heterogeneity through differentiated TME features whose characterization translates into complex and highly contextualized data fusions. New targeted computational inferences are here needed.

Saliency maps were also addressed to assign phenotypic relevance to the brain regions and delineate predictively the features value for an assessment of the risk of relapse, again requiring consideration of contextual characteristics to be suitably represented in computational models.

We then addressed an interesting space, atlas-driven radiomics, which complements the traditional targeted radiomics. The latter is based on consolidated main steps which include building tumor segmentations, extracting features from ROI and classifying patients based on them. As an alternative, we showed that the use of brain atlases allows the design of interpretable templates that can become implementable tools depending on their definition and how their structures adapt to the various contexts, brain regions in our case.

We illustrated the potential use of such templates with GBM patients, and we easily foresee their generalizability to other brain cancers, always with the possibility of visualizing parcellation maps through applied computational tools, i.e., biclustering or similar. Concerning the value of this type of analysis for clinical scopes, the associations between brain regions and radiomic features can become more significant with the scale of the study, in which case features distributions classified by brain regions and patients may become informative for stratification purposes. Finally, the characterization that the template maps offer is useful for monitoring the disease evolution, and in such regard, concepts like imaging habitats and saliency are extremely informative and deserve further investigation.

## Supplementary information

**Additional file 1:** SM_Allregions file: list of atlas parcellation- derived brain regions.

**Additional file 2:** SM_ALLfeatures file: list of extracted features across all MRI modalities.

## Data Availability

Not applicable.

## Abbreviations

GBM: Glioblastoma multiforme
CT: Computed tomography
MRI: Magnetic resonance imaging
RWD: Real-world data
TME: Tumor microenvironment
ROI: Regions of interest
GLCM: Gray level co-occurrence matrix
ML: Machine learning
DL: Deep learning
TCIA: The cancer imaging archive
XAI: Explainable artificial intelligence
TP: Tumor progression
PsP: Pseudo-progression
dMRI: Diffusion MRI
sMRI: structural MRI
Ivy GAP: Ivy glioblastoma atlas project
NITRC: NeuroImaging tools and resources collaboratory
TCGA-: GBM The cancer genome atlas glioblastoma multiforme
CSF: Cerebrospinal fluid
DWI: Diffusion-weighted imaging
IT: Information technology

## References

[1] Poon MTC, Sudlow CLM, Figueroa JD (2020). Longer-term (≥ 2 years) survival in patients with glioblastoma in population-based studies pre- and post-2005: a systematic review and meta-analysis. Sci Rep.

[2] Crespo I, Vital AL, Gonzalez-Tablas M (2015). Molecular and genomic alterations in glioblastoma multiforme. Amer J Path.

[3] Li Z-H, Guan Y-L, Zhang G-B (2021). Genomic analysis of glioblastoma multiforme reveals a key transcription factor signature relevant to prognosis and the immune processes. Front Oncol..

[4] Sakthikumar S, Roy A, Haseeb L (2020). Whole-genome sequencing of glioblastoma reveals enrichment of non-coding constraint mutations in known and novel genes. Genome Biol.

[5] Cui X, Wang Q, Zhou J, at al. (2021). Single-cell transcriptomics of glioblastoma reveals a unique tumor microenvironment and potential immunotherapeutic target against tumor-associated macrophage. Front Oncol.

[6] Couturier CP, Ayyadhury S, Le PU (2020). Single-cell RNA-seq reveals that glioblastoma recapitulates a normal neurodevelopmental hierarchy. Nat Commun.

[7] Bikfalvi A, da Costa CA, Avril T (2023). Challenges in glioblastoma research: focus on the tumor microenvironment. Trends Cancer.

[8] Ravi VM, Will P, Kueckelhaus J (2022). Spatially resolved multi-omics deciphers bidirectional tumor-host interdependence in glioblastoma. Cancer Cell.

[9] Ding H, Wu C, Liao N (2021). Radiomics in oncology: a 10-Year bibliometric analysis. Front Oncol.

[10] van Timmeren J, Cester D, Tanadini-Lang S (2020). Radiomics in medical imaging—“how-to” guide and critical reflection. Ins Imaging.

[11] Rogers W, Thulasi Seetha S, Refaee TAG (2020). Radiomics: from qualitative to quantitative imaging. Br J Radiol.

[12] Bae S, An C, Ahn SS (2020). Robust performance of deep learning for distinguishing glioblastoma from single brain metastasis using radiomic features: model development and validation. Sci Rep.

[13] Fu J, Singhrao K, Zhong X (2021). An Automatic deep learning-based workflow for glioblastoma survival prediction using preoperative multimodal MR images: a feasibility study. Adv Radiat Oncol.

[14] Le NQK, Hung TNK, Do DT (2021). Radiomics-based machine learning model for efficiently classifying transcriptome subtypes in glioblastoma patients from MRI. Comp Biol Med..

[15] Yan J, Zhao Y, Chen Y (2021). Deep learning features from diffusion tensor imaging improve glioma stratification and identify risk groups with distinct molecular pathway activities. EBioMedicine.

[16] Liu Z, Jiang Z, Meng L (2021). Handcrafted and deep learning-based radiomic models can distinguish GBM from brain metastasis. J Oncol.

[17] Lao J, Chen Y, Li ZC (2017). A deep learning-based radiomics model for prediction of survival in glioblastoma multiforme. Sci Rep.

[18] Han W, Qin L, Bay C (2020). Deep transfer learning and radiomics feature prediction of survival of patients with high-grade gliomas. Amer J Neuroradiol.

[19] Afshar P, Mohammadi A, Plataniotis KN (2019). From handcrafted to deep-learning-based cancer radiomics: challenges and opportunities. IEEE Signal Proc Magaz.

[20] Papanikolaou N, Matos C, Koh DM (2020). How to develop a meaningful radiomic signature for clinical use in oncologic patients. Cancer Imag.

[21] Huang EP, O’Connor JPB, McShane LM (2023). Criteria for the translation of radiomics into clinically useful tests. Nat Rev Clin Oncol.

[22] Clark K, Vendt B, Smith K (2013). The cancer imaging archive (TCIA): maintaining and operating a Public Information Repository. J Digi Imaging.

[23] Arita H, Kinoshita M, Kawaguchi A (2018). Lesion location implemented magnetic resonance imaging radiomics for predicting *IDH* and *TERT* promoter mutations in grade II/III gliomas. Sci Rep.

[24] Arimura H, Soufi M, Ninomiya K (2018). Potentials of radiomics for cancer diagnosis and treatment in comparison with computer-aided diagnosis. Radiol Phys Technol.

[25] Bodalal Z, Trebeschi S, Beets-Tan R (2018). Radiomics: a critical step towards integrated healthcare. Ins Imaging.

[26] Papadimitroulas P, Brocki L, Christopher Chung N (2021). Artificial intelligence: deep learning in oncological radiomics and challenges of interpretability and data harmonization. Phys Med.

[27] Severn C, Suresh K, Görg C (2022). A Pipeline for the implementation and visualization of explainable machine learning for medical imaging using radiomics features. Sensors.

[28] Varriano G, Guerriero P, Santone A (2022). Explainability of radiomics through formal methods. Comp Meth Progr Biomed.

[29] Chen H, Gomez C, Huang CM (2022). Explainable medical imaging AI needs human-centered design: guidelines and evidence from a systematic review. npj Digit Med.

[30] Ladbury C, Zarinshenas R, Semwal H (2022). Utilization of model-agnostic explainable artificial intelligence frameworks in oncology: a narrative review. Transl Cancer Res.

[31] Kim AR, Choi KS, Kim MS (2021). Absolute quantification of tumor-infiltrating immune cells in high-grade glioma identifies prognostic and radiomics values. Cancer Immunol Immunother.

[32] Li ZZ, Liu PF, AnTT (2021). Construction of a prognostic immune signature for lower grade glioma that can be recognized by MRI radiomics features to predict survival in LGG patients. Translat Oncol.

[33] Booth TC, Ashkan K, Brazil L (2016). Tumour progression or pseudoprogression? a review of post-treatment radiological appearances of glioblastoma. Clin Radiol.

[34] Kocher M, Ruge MI, Galldiks N (2020). Applications of radiomics and machine learning for radiotherapy of malignant brain tumors. Strahlenther Onkol.

[35] Sala E, Mema E, Himoto Y (2017). Unravelling tumour heterogeneity using next-generation imaging: radiomics, radiogenomics, and habitat imaging. Clin Radiol.

[36] Waqar M, Van Houdt PJ, Hessen E (2022). Visualising spatial heterogeneity in glioblastoma using imaging habitats. Front Oncol.

[37] Zhang L, Wang Y, Peng Z (2022). The progress of multimodal imaging combination and subregion based radiomics research of cancers. Int J Biol Sci.

[38] Tar PD, Thacker NA, Babur M (2022). Imaging of tumors enables high confidence sub-regional assessment of response to therapy. Cancers.

[39] Bernatowicz K, Grussu F, Ligero M (2021). Robust imaging habitat computation using voxel-wise radiomics features. Sci Rep.

[40] Verma R, Correa R, Hill VB (2020). Tumor habitat-derived radiomic features at pretreatment MRI that are prognostic for progression-free survival in glioblastoma are associated with key morphologic attributes at histopathologic examination: a feasibility study. Radiol Artif Intell.

[41] Chiu F-Y, Yen Y (2022). Efficient radiomics-based classification of multi-parametric MR images to identify volumetric habitats and signatures in glioblastoma: a machine learning approach. Cancers.

[42] Chaddad A, Kucharczyk MJ, Daniel P (2019). Radiomics in glioblastoma: current status and challenges facing clinical implementation. Front Oncol.

[43] Mandal AS, Romero-Garcia R, Seidlitz J (2021). Lesion covariance networks reveal proposed origins and pathways of diffuse gliomas. Brain Commum.

[44] Fathi Kazerooni A, Bagley SJ, Akbari H (2021). Applications of radiomics and radiogenomics in high-grade gliomas in the era of precision medicine. Cancers.

[45] Fathi Kazerooni A, Saxena S, Toorens E (2022). Clinical measures, radiomics, and genomics offer synergistic value in AI-based prediction of overall survival in patients with glioblastoma. Sci Rep.

[46] Shui L, Ren H, Yang X (2021). The era of radiogenomics in precision medicine: an emerging approach to support diagnosis, treatment decisions, and prognostication in oncology. Front Oncol.

[47] Lo Gullo R, Daimiel I, Morris EA (2020). Combining molecular and imaging metrics in cancer: radiogenomics. Ins Imaging.

[48] Chow D, Chang P, Weinberg BD (2018). Imaging genetic heterogeneity in glioblastoma and other glial tumors: review of current methods and future directions. AJR Amer J Roentgenol.

[49] Fathi Kazerooni A, Bakas S, Saligheh Rad H (2020). Imaging signatures of glioblastoma molecular characteristics: a radiogenomics review. J Magn Reson Imaging.

[50] Liu Z, Wu K, Wu B (2021). Imaging genomics for accurate diagnosis and treatment of tumors: a cutting edge overview. Biomed  Pharmacoth.

[51] Coupet M, Urruty T, Leelanupab T (2022). A multi-sequences MRI deep framework study applied to glioma classfication. Multimed Tools Appl.

[52] Taghizadeh S, Labuda C, Yang CC (2019). Optimizing MRI sequences and images for MRI-based stereotactic radiosurgery treatment planning. Rep Pract Oncol Radiother.

[53] Lv W, Ashrafinia S, Ma J (2020). Multi-level multi-modality fusion radiomics: application to PET and CT imaging for prognostication of Head and neck cancer. IEEE J Biomed Health Inform.

[54] Amini M, Nazari M, Shiri I (2021). Multi-level multi-modality (PET and CT) fusion radiomics: prognostic modeling for non-small cell lung carcinoma. Phys Med Biol.

[55] Manafi-Farid R, Askari E, Shiri I (2022). [^18^F]FDG-PET/CT Radiomics and artificial intelligence in lung cancer: technical aspects and potential clinical applications. Sem Nucl Med.

[56] Li Z, Holzgreve A, Unterrainer LM (2023). Combination of pre-treatment dynamic [^18^F]FET PET radiomics and conventional clinical parameters for the survival stratification in patients with IDH-wildtype glioblastoma. Eur J Nucl Med Mol Imaging.

[57] Sakai Y, Yang C, Kihira S (2020). MRI Radiomic features to predict IDH1 mutation status in gliomas: a machine learning approach using gradient tree boosting. Int J Mol Sci.

[58] Kline A, Wang H, Li Y, Dennis S (2022). Multimodal machine learning in precision health: a scoping review. NPJ Digit Med.

[59] Mansouri N, Balvay D, Zenteno O (2023). Machine learning of multi-modal tumor imaging reveals trajectories of response to precision treatment. Cancers.

[60] MacFadyen C, Duraiswamy A, Harris-Birtill D (2023). Classification of hyper-scale multimodal imaging datasets. medRxiv.

[61] Wang T, Lei Y, Fu Y (2021). A review on medical imaging synthesis using deep learning and its clinical applications. J Appl Clin Med Phys.

[62] Yu B, Wang Y, Wang L (2020). Medical image synthesis via deep learning. Adv Exp Med Biol.

[63] Dai X, Lei Y, Fu Y (2020). Multimodal MRI synthesis using unified generative adversarial networks. Med Phys.

[64] Li W, Li Y, Qin W (2020). Magnetic resonance image (MRI) synthesis from brain computed tomography (CT) images based on deep learning methods for magnetic resonance (MR)-guided radiotherapy. Quant Imaging Med Surg.

[65] Li W, Kazemifar S, Bai T (2021). Synthesizing CT images from MR images with deep learning: model generalization for different datasets through transfer learning. Biomed Phys Eng Express.

[66] Tomaszewski MR, Gillies RJ (2021). The biological meaning of radiomic features (Erratum for: Radiology. 2021;298(3):505–516). Radiology..

[67] Zhang Z, Yang J, Ho A (2018). A predictive model for distinguishing radiation necrosis from tumour progression after gamma knife radiosurgery based on radiomic features from MR images (Erratum in: Eur Radiol. 2018 Mar 14; PMID: 29178031; PMCID: PMC6036915). Eur Radiol.

[68] Delery W, Savjani RR (2023). Radiation necrosis versus tumor progression: the path toward an optimal discriminator. Radiol Imaging Cancer.

[69] Peng L, Parekh V, Huang P (2018). Distinguishing true progression from radionecrosis after stereotactic radiation therapy for brain metastases with machine learning and radiomics. Int J Radiat Oncol Biol Phys.

[70] Chen X, Parekh VS, Peng L (2021). Multiparametric radiomic tissue signature and machine learning for distinguishing radiation necrosis from tumor progression after stereotactic radiosurgery. Neuro-Oncol Adv.

[71] Park YW, Choi D, Park JE (2021). Differentiation of recurrent glioblastoma from radiation necrosis using diffusion radiomics with machine learning model development and external validation. Sci Rep.

[72] Zhang Q, Cao J, Zhang J (2019). Differentiation of recurrence from radiation necrosis in gliomas based on the radiomics of combinational features and multimodality MRI images. Comput Math Methods Med.

[73] Correa R, Lei Q, Chen J et al. Lesion-habitat radiomics to distinguish radiation necrosis from tumor recurrence on post-treatment MRI in metastatic brain tumors. Proc. SPIE (Medical Imaging 2020: Computer-Aided Diagnosis) 2020; 1131430. 10.1117/12.2551393.

[74] Eickhoff SB, Yeo BTT, Genon S (2018). Imaging-based parcellations of the human brain. Nat Rev Neurosci.

[75] Dworetsky A, Seitzman BA, Adeyemo B (2021). Probabilistic mapping of human functional brain networks identifies regions of high group consensus. NeuroImage.

[76] Liu T (2011). A few thoughts on brain ROIs. Brain Imaging Behav.

[77] Škoch A, Rehák Bučková B, Mareš J (2022). Human brain structural connectivity matrices–ready for modelling. Sci Data.

[78] Markello RD, Hansen JY, Liu ZQ (2022). Neuromaps: structural and functional interpretation of brain maps. Nat Methods.

[79] Lawrence RM, Bridgeford EW, Myers PE (2021). Standardizing human brain parcellations. Sci Data.

[80] Zhao M, Liu Y, Ding G (2021). Online database for brain cancer-implicated genes: exploring the subtype-specific mechanisms of brain cancer. BMC Genomics.

[81] Puchalski RB, Shah N, Miller J (2018). An anatomic transcriptional atlas of human glioblastoma. Science.

[82] Pati S, Verma R, Akbari H (2020). Reproducibility analysis of multi-institutional paired expert annotations and radiomic features of the Ivy glioblastoma atlas project (Ivy GAP) dataset. Med Phys.

[83] Van Essen DC, Smith SM, Barch DM (2013). The WU-Minn human connectome project: an overview. NeuroImage.

[84] Alemán-Gómez Y, Griffa A, Houde JC (2022). A multi-scale probabilistic atlas of the human connectome. Sci Data.

[85] Mandal AS, Romero-Garcia R, Hart MG (2020). Genetic, cellular, and connectomic characterization of the brain regions commonly plagued by glioma. Brain.

[86] Mandal AS, Romero-Garcia R, Seidlitz J (2021). Lesion covariance networks reveal proposed origins and pathways of diffuse gliomas. Brain Commun.

[87] Neftel C, Laffy J, Filbin MG (2019). An integrative model of Cellular states, plasticity, and genetics for glioblastoma. Cell.

[88] Kong NW, Gibb WR, Tate MC (2016). Neuroplasticity: insights from patients harboring gliomas. Neural Plast.

[89] Lv K, Cao X, Wang R (2022). Neuroplasticity of glioma patients: brain structure and topological network. Front Neurol..

[90] van Griethuysen JJM, Fedorov A, Parmar C (2017). Computational radiomics system to decode the radiographic phenotype. Cancer Res.

[91] Pedregosa F, Varoquaux G, Gramfort A (2011). Scikit-learn: machine learning in Python. JMLR.

